# Impact of consumer information capability on green consumption intention: the role of green trust and media publicity

**DOI:** 10.3389/fpsyg.2023.1247479

**Published:** 2023-10-09

**Authors:** Gong-Li Luo, Hao Zheng, Yan-Lu Guo

**Affiliations:** College of Economics and Management, Shandong University of Science and Technology, Qingdao, China

**Keywords:** information capability, green trust, media publicity, green consumption intention, information asymmetry theory

## Abstract

In the context of the digital information era, consumers’ information capability plays a crucial role in shaping their green consumption intention. To delve deeper into the dynamics of how information capability influences consumers’ green consumption intention, this study constructs a theoretical model grounded in information asymmetry theory and cognitive bias theory. Additionally, the mediating role of green trust and the moderating role of media publicity are incorporated to comprehensively investigate the influence mechanism of information capability on consumers’ green consumption intention. Empirical analysis is conducted based on data collected through a questionnaire survey. The findings of this study reveal that information capability exerts a partially mediating effect on consumers’ green consumption intention. Specifically, information capability directly influences consumers’ green consumption intention, and it also indirectly impacts their green consumption intention through its influence on green trust. Furthermore, media publicity positively moderates the relationship between information capability and consumers’ green consumption intention. This research contributes to the existing literature by enhancing our understanding of the influence pathway leading to green consumption intention. Its insights offer valuable implications for promoting green consumption and advancing sustainable development initiatives.

## 1. Introduction

In light of the burgeoning popularity of network technology and the rapid advancements in emerging technologies such as big data and cloud computing, the world has entered an era defined by digital information. This era has not only broadened the spectrum of consumption methods but has also provided consumers with an expanded array of choices (Bartschat et al., 2022). However, the unsound and irrational consumption patterns exhibited by consumers, despite being a driving force for economic growth, have inflicted long-standing harm upon the ecological environment. Consequently, addressing this issue necessitates a shift in consumer behavior, wherein consumption patterns align with ecological civilization and sustainable development, thereby minimizing environmental damage and carbon emissions while still satisfying demands. The notion of green consumption, first introduced in 1987, has garnered escalating attention and recognition as a significant constituent of the global sustainable development movement (Akhtar et al., 2020). The twentieth report emphasizes the promotion of green development, the attainment of harmonious coexistence between humanity and nature, the advocacy for green consumption, and the cultivation of green and low-carbon modes of production and lifestyle. Consequently, academia is currently deeply invested in exploring the underlying mechanisms behind the formation of green consumption intentions and promoting the active adoption of green consumption practices by consumers. Moreover, in the face of the overwhelming abundance of information, the ability to swiftly and accurately extract and filter pertinent and useful information assumes paramount importance, placing heightened demands on information capability.

Certain scholars have highlighted that consumers’ inclination toward engaging in green consumption is somewhat constrained by their level of information (Luan et al., 2023). This observation revolves around two fundamental aspects. Firstly, information, acting as a catalyst for green consumption (Ran et al., 2022), reveals that variances in consumers’ information aptitude may impede the progress of the green market. The extent of consumers’ knowledge concerning green products and the available purchasing channels exerts an influence on the development of green consumption. Secondly, the actualization of purchasing behaviors related to green products necessitates a genuine green value inherent within the products (Xu et al., 2022). The green value and product information serve as crucial determinants in consumers’ decision-making and purchase choices. Regrettably, there exists an asymmetry in the availability of such information, as the suppliers possess a better understanding of the organic green attributes of their products, while consumers face challenges in identifying these attributes. Consequently, to enhance consumers’ willingness to embrace green products, the key lies in augmenting consumers’ information capability and fostering the robust advancement of the green consumption market. Regrettably, extant scholarly investigations appear to have predominantly overlooked this particular concern. The prevailing emphasis has been on information provisioning, encompassing facets like eco-labeling, traceability information, and traceability systems (Brian et al., 2015; Wu et al., 2015; Di et al., 2019), all geared toward mitigating the repercussions of information asymmetry and consequentially enhancing product efficacy. Conversely, relatively limited scrutiny has been devoted to the information demand aspect, with scholars paying less attention and discourse to the influence of consumers’ information capability on their consumption proclivities. This research lacuna provides the impetus for the present study.

Nowadays, in the era of the information explosion, we are receiving information from all directions all the time, and we are wrapped up in a huge amount of information. Nonetheless, within the framework of information asymmetry, discerning consumers frequently opt against placing trust, owing to the potential for deceit and other malevolent actions on the part of information disseminators (Michael and Edi, 1973). Thus, fostering the salubrious progression of the green consumer market hinges upon addressing information asymmetry and bolstering consumer confidence. Concurrently, trust holds significant importance as a determinant in consumer decision-making processes (Niedermeier et al., 2020). It serves as a fundamental precondition for consumers when engaging in consumption activities. Given that green products possess distinct characteristics that set them apart from other products, consumers must assess both the intrinsic product value and the associated green value before opting for green products, thereby fostering trust in green consumption. Consequently, this study introduces and examines the potential mediating role of green trust in investigating the influence of information capability on green consumption intentions. Additionally, individual behavioral intentions are not solely driven by normative constraints but are also subject to external environmental influences (Gabi, 2020). Media publicity, for instance, have the capacity to shape consumers’ consumption behaviors. In the digital era, media propaganda assumes a pivotal role, and within the realm of green consumption, the impact of information capability may vary under the sway of media propaganda. For instance, companies leverage media platforms to promote green products and establish green brand identities, thereby augmenting the influence of information capability on green consumption intentions. Building upon this foundation, this study incorporates media publicity into the research framework to explore its moderating effect on the relationship between information capability and green consumption intentions.

To summarize, this study adopts information capability as the focal point and in-corporates green trust, media publicity, and consumers’ green consumption intentions into the research framework. It aims to investigate the influence of information capability on consumers’ green consumption intentions and the underlying pathways of in-fluence. This paper introduces several possible innovations. Firstly, by focusing on in-formation capability as the primary research subject, it offers a new perspective on the matter of green consumption and broadens the scope of green consumption research. Secondly, in addition to exploring the direct effects, this study further incorporates green trust as mediating variables and media publicity as moderating variables, respectively. By doing so, it endeavors to examine the intricate mechanisms through which information capability affects consumers’ green consumption intentions in the information era, thus offering a more comprehensive and exquisite understanding of the phenomenon.

## 2. Literature review

### 2.1. Information capability

Paul Zurkowski introduced the concept of information capability, defining it as the aptitude of individuals to utilize information resources in their productive lives (Behrens, 1994). The Information Literacy Framework for Higher Education in the United States asserts that information literacy encompasses an awareness of information needs and the capability to acquire, evaluate, integrate, and utilize information (Nancy, 2015). McClure posits that individuals with a high level of information capability should possess the following attributes: knowledge of information systems; the capacity to access, analyze, organize, and evaluate information; comprehension of the value, function, and role of information; and the ability to employ information in problem-solving endeavors (Mcclure, 1994). Therefore, information capability includes not only the acquisition of information, but also the capability to judge and process information, as well as the capability to utilize information. Therefore, building upon prior research (Wu Q. et al., 2022), this paper delineates information capability into three dimensions: information cognitive capability, information acquisition capability, and information sharing capability. Information cognitive capability pertains to consumers’ level of knowledge regarding the functionalities or values associated with green products. Information acquisition capability pertains to consumers’ ability to precisely define their information requirements and effectively gather and obtain information through various channels. Lastly, information sharing capability signifies consumers’ ability to proficiently employ information, as well as their capacity to share and communicate information with others.

The study of information capability is commonly grounded in the measurement of this construct. Scholars have employed various methods to measure information capability, resulting in a diverse range of approaches. For instance, Khatskevich (2022) investigated a class of methods employed in tackling information evaluation problems, thereby enabling the assessment of users’ information capability. Zrnec et al. (2022) utilized information quality as a tool to gage users’ capacity to discern misinformation. Similarly, Allari et al. (2022) measured the information literacy capability of Jordanian nursing students. Furthermore, literature contains studies that examine the impact of information capability on consumer behavior from a singular perspective. Notably, cognitive capability has been shown to influence consumers’ willingness to purchase biofortified foods (Razzaq et al., 2021). Moreover, disparities in information accessibility have demonstrated a significant positive influence on farmers’ green behavior (Lv and Li, 2023). Additionally, the act of information sharing by leaders has been observed to affect subordinates’ proactive behavior (Liu et al., 2022).

While a considerable body of literature focuses on designing scales to measure information capability, fewer studies delve into the relationship between information capability and green consumption intentions as a pivotal antecedent variable. Additionally, the majority of scholars tend to examine consumer behavior through the lens of individual variables, with a limited number of researchers comprehensively considering the concept of information capability and conducting in-depth analyses of its influence on consumers’ green consumption intentions.

### 2.2. Green consumption intention

Green consumption entails individual consumers’ proactive endeavor to acquire products or services imbued with environmental values (Otterbring et al., 2023). It seeks to diminish resource consumption and mitigate pollution emissions throughout the consumption process, thereby fostering environmental protection and resource conservation. In the realm of consumer behavior research, consumption intention is widely acknowledged as a potent predictor of actual consumption behavior (Tong et al., 2023). Scholars have explored various influencing factors that shape green consumption intentions from diverse perspectives. Firstly, certain researchers have examined the impact of demographic characteristics, such as gender and age, on green consumption intentions (Septianto and Kemper, 2021; Yang et al., 2021). Secondly, psychological factors, including attitude and environmental knowledge, have been scrutinized for their influence on green consumption intentions (Chaihanchanchai and Anantachart, 2022; Hossain et al., 2022). Moreover, scholars have investigated the effects of external factors, such as green advertising, social norms, and policies, on green consumption intentions (Ramesh et al., 2019; Siti et al., 2020; Feng et al., 2022).

In general, numerous scholars have examined the determinants of green consumption intentions from three main perspectives: demographic characteristics, psychological factors, and external factors. They have further developed conceptual models to explore the effects of these factors on green consumption intentions. However, there remains a dearth of research that approaches the topic from the perspective of information capability, necessitating further exploration in this area.

## 3. Theoretical models and basic hypotheses

### 3.1. Information capability and green consumption intention

As cognitive beings, humans possess the ability to transform ontological information concerning the external world, which is influenced by the intrinsic nature of things, into diverse forms of syntactic, semantic, and pragmatic information. This process is facilitated through their perception, comprehension, and value judgments (Courtade et al., 2017). Consequently, consumers engage in the assimilation, analysis, and contemplation of acquired knowledge and information, subsequently making behavioral decisions based on the outcomes of this information processing. The existence of cognitive biases among individual consumers, known as consumer cognitive biases, highlights the presence of inherent limitations in their capacity to effectively process and interpret information (Kahneman and Tversky, 1979), information handling and processing occurs throughout an individual’s behavioral decision-making process, while illustrating the information plays a crucial role in the process of individual behavior change.

Social cognitive theory posits that individual behavior is influenced by the interplay of three components: cognition, behavior, and the environment (Albert, 2012). Green consumption, characterized by minimizing adverse environmental impacts (Li et al., 2020), aims to safeguard the ecological environment throughout the stages of purchasing, utilizing, and disposing of goods. This behavior is influenced by cognitive processes. Simultaneously, the production of green products incorporates environmentally friendly attributes, thereby mitigating environmental impacts. The use of green products, in turn, benefits not only the environment but also human health, making it a self-interested and altruistic endeavor. Since individual behavioral decisions are contingent upon the information processing mechanisms employed by cognitive systems (Adrian and Ben, 2013), it is important to acknowledge that consumers possess limitations in information cognition and recognition. However, when consumers exhibit a heightened level of information cognition, they develop a clearer understanding of green consumption, as well as the benefits and values associated with green products. This heightened awareness subsequently enhances their willingness to adopt the green consumption model.

Secondly, consumers’ information access capability significantly impacts the quality of information collection and processing. Green consumption represents a novel consumption model that caters to individual needs while prioritizing the overall welfare of society (Hung et al., 2019). This consumption model is characterized by a certain premium. According to information asymmetry theory (Cavaliere and Crea, 2021), each party involved in a transaction possesses varying levels of information, and the party with less information strives to acquire information from the other party. Consumers evaluate the perceived green value of a product through intrinsic cues, such as raw materials, as well as extrinsic information, such as packaging and brand image. As “rational individuals,” consumers take into account not only the societal benefits of green products but also the potential risks associated with their premium pricing. When consumers have enhanced access to information, they possess greater resources and enjoy an information advantage. Consequently, they can swiftly comprehend the value and advantages offered by green products, thus increasing their willingness to pay a premium and subsequently bolstering their inclination to engage in green consumption (Jin et al., 2023).

Thirdly, rational behavior theory posits that information collection and processing can shape individuals’ behavioral intentions by adjusting subjective norms, thereby influencing their behavioral decisions (Muddasar et al., 2020). Through the sharing, exchange, or dissemination of information, consumers with a high capability for information sharing actively propagate the benefits of green consumption and guide green consumption behavior. As consumers undergo the process of making behavioral decisions, they are influenced by pressures from friends, relatives, and evaluations from others, which ultimately shape subjective norms and related factors (Manoela and Edvan, 2020). These subjective norms, among other influences, can positively impact the willingness to purchase green products. Consequently, this can help overcome challenges such as the resistance toward accepting premiums associated with green products. Moreover, this process contributes to consumers’ mastery and utilization of green products, ultimately enhancing their willingness to engage in green consumption.

In summary, this paper posits the following hypothesis:

*H1*: Information capability exerts a positive influence on consumers’ green consumption intention.

### 3.2. The mediating role of green trust

Green trust refers to the beliefs and expectations formed by consumers based on the competence, reliability, and goodwill of the green product itself and its producer, which subsequently influences their willingness to trust the company and the product (Chen, 2010). Trust serves as an essential public good for maintaining human social order and serves as the foundation for all economic activities. Trust may stem from innate human instincts or individual preferences developed through natural evolution. Alternatively, it may be grounded in “rational calculation” of an object’s future actions (Laura et al., 2016). Thus, trust exhibits both “emotional” and “rational” characteristics. According to information processing theory, consumers’ decision-making processes are typically categorized into two distinct systems and modes: rational (cognitive) analysis and intuitive (emotional) inspiration (Zhao et al., 2011). In certain instances, consumers’ green trust may originate directly from intuition and emotion. However, in situations involving high stakes, complexity, and psychological distance, rational cognitive analysis plays a pivotal role in trust formation. Green food, being a functional product closely associated with consumers’ health and safety, entails specialized and intricate product knowledge and often commands a certain premium. As a result, consumers tend to rely on the rational cognitive system to process information about these products and form judgments regarding their trustworthiness. Therefore, the concept of consumers’ green trust in this paper aligns with a “rational” form of trust.

In general, consumers with high information capability possess a greater understanding of the ecological challenges stemming from rapid economic growth, such as environmental degradation and resource scarcity. They are more knowledgeable about the environmental benefits and health advantages associated with green consumption, which cultivates trust in green products and green consumption. Simultaneously, information asymmetry creates a profound sense of uncertainty among consumers, triggering anxiety during decision-making processes. To mitigate risks, consumers actively seek out product-related information from various channels, including product brands, certifications, origin details, sales channels, and consumer reviews. This process of “information acquisition” furnishes consumers’ rational cognitive system with analytical materials and decision-making foundations, thus facilitating the development of trust. Previous research has also demonstrated that information capability can impact trust (Nisha et al., 2016). When consumers possess comprehensive knowledge about the merits and values of green products, coupled with abundant and high-quality information resources, their level of green trust tends to be stronger.

Moreover, consumer green trust plays a significant role in shaping consumers’ adoption of green consumption and altering their consumption patterns. Carsten et al. demonstrated that a higher level of trust in eco-labels correlates with an increased likelihood of purchasing organic food and engaging in green consumption (Carsten et al., 2014). Similarly, Martin conducted a case study on the Danish organic food system and observed that trustworthiness holds particular significance within this system, as higher levels of consumer trust are associated with stronger inclinations toward green consumption (Martin, 2015). As a novel consumption model, green consumption benefits from the enhancement of consumer beliefs regarding products and companies, evoking positive emotions and, consequently, amplifying consumers’ willingness to engage in green consumption (Wang and Wangt, 2022). Trust exercises a pivotal influence on consumers’ selection and implementation of green consumption behaviors, with individuals exhibiting higher levels of trust displaying greater intentions for green consumption. Consequently, this paper proposes the following hypothesis.

*H2*: Green trust serves as a mediating mechanism in the relationship between information capability and green consumption intention.

### 3.3. The moderating effect of media publicity

According to social cognitive theory, individual behavioral choices are influenced not only by internal factors such as cognition but also by the external environment. Media, as a conduit of information dissemination, has a significant impact on individuals’ access to information and their engagement in social activities. With the advent of Internet technology, media publicity have emerged as a crucial means through which individuals acquire information (Dongfang, 2021). The perceived healthiness and safety of green products serve as fundamental prerequisites for consumers to engage in environmentally conscious purchases. Additionally, product authenticity plays a crucial role in alleviating consumer apprehensions (Tony et al., 2022). Companies leverage media platforms to promote the green attributes of their products, thus enhancing the perceived authenticity of their green brands. When consumers consider purchasing green products, they associate the continuous environmental benefits and green value of the product with its media-driven promotion. Consequently, this reinforcement of trust in the product’s green attributes motivates consumers with high information capability to exhibit a greater willingness to make purchase decisions. Conversely, weak media publicity surrounding a product, while not altering the objective fact that the product is environmentally beneficial, may instill doubt regarding the authenticity of its green attributes. Consequently, this reduction in perceived control over the purchase behavior of the green product may diminish consumers’ willingness to engage with it. Individuals with high information capability may be particularly susceptible to this influence, leading to a decrease in their propensity to purchase the green product.

*H3*: Media publicity exerts a positive moderating effect on the relationship between information capability and green consumption intention.

**Figure 1:**
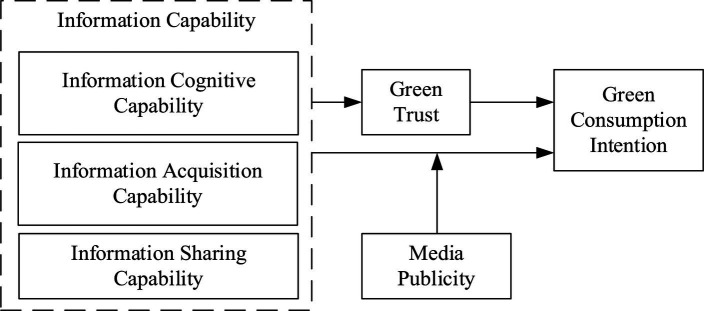
Hypothesis framework.

In summary, this paper presents the research model depicted in Figure 1, which illustrates the conceptual framework for the study.

## 4. Results

### 4.1. Scale measurement and descriptive statistics

In this research paper, we devised a set of demographic characteristics factors, namely gender, age, academic qualifications, monthly salary, and career, with the objective of examining the presence of variability within these factors. Additionally, apart from the aforementioned demographic characteristics, our questionnaire incorporated six variables: information cognitive capability, information acquisition capability, information sharing capability, green trust, media publicity, and green consumption intention. Data pertaining to these variables were gathered utilizing a 5-point Likert scale methodology. Information cognitive capability pertains to the extent of individuals’ perception regarding the role or value of external information relevant to the study, primarily drawing upon Zhao’s scale (Zhao et al., 2014). Information acquisition capability reflects the sufficiency of consumer information acquisition, with information acquisition channels encompassing the Internet, television and radio, newspapers and magazines, exchange with relatives and friends, and sales staff recommendations. Enhanced information acquisition capability indicates more comprehensive information gathering and facilitates the reduction of information asymmetry, primarily referencing Wu’s scale (Wu S. et al., 2022). Information sharing capability refers to the aptitude to share and exchange information with others, thereby enabling the dissemination of information value. This aspect was predominantly informed by Yudi’s scale (Yudi et al., 2020). Green trust, as a construct, signifies the belief system and identification of suitable variables for gaging the level of trust among individuals, primarily drawing upon Chen’s trust level scale (Chen, 2010). Media publicity denotes companies’ utilization of media platforms to showcase the green attributes of their products, thus establishing their green brand image. This dimension primarily draws upon Kaman’s scale (Kaman, 2010). Finally, green consumption intention encapsulates consumers’ willingness to adopt behaviors that mitigate the adverse environmental effects of consumption and is considered a robust predictor of green purchasing behavior. This construct was adapted by incorporating elements from So-Yun’s scale (So-Yun et al., 2012) (see Table 1).

**Table 1:** Item scale summaries.

Variable	Number	Items	References
Information cognitive capability	IC1	I know the difference between green food, pollution-free food, organic food	Zhao et al. (2014)
	IC2	Green consumption is good for the environment	
	IC3	I know that green food, pollution-free food, and organic food are better for your health.	
Information acquisition capability	IA1	Getting the information I need in a timely manner is easy for me.	Wu S. et al. (2022)
	IA1	I was able to access a larger amount of information	
	IA1	The information I’ve gathered is true and valid	
Information sharing capability	IS1	I often discuss the benefits of green consumption with my friends	Yudi et al. (2020)
	IS1	Exchanging price information on green products with others is easy for me	
	IS1	I often talk to my family and friends about ways to buy green products	
Green trust	GT1	The production process of green products meets green requirements	Chen (2010)
	GT1	I believe that green products are priced in line with green values	
	GT1	I believe that green product reprocessing reduces environmental damage.	
Media publicity	MP1	The media publicity has done a good job of publicizing the green attributes of green products	Kaman (2010)
	MP1	The media publicity is well placed to publicize the environmental benefits of green consumption	
	MP1	I think the green products promoted by the media publicity have authenticity	
Green consumption intention	GCI1	I plan to go green in the near future	So-Yun et al. (2012)
	GCI1	I’m willing to pay more for green products	
	GCI1	Faced with a product with the same features, I would choose the product with green attributes	

In this study, we primarily employed an online data collection methodology, with data acquisition facilitated through a questionnaire survey conducted on the “Questionstar” website.^1^ To mitigate potential sampling bias associated with concentrating responses from a single city, and to ensure that our findings are not confined to a specific geographic region, we meticulously considered this aspect during questionnaire distribution. The questionnaires were disseminated across various cities spanning different regions, each characterized by distinctive attributes including economic development levels and educational backgrounds. This deliberate approach was implemented to, in part, attenuate the influence of data from regions that closely resembled each other, thus bolstering the generalizability and robustness of our research outcomes. Through the dissemination of these questionnaires, a total of 653 responses were received. Subsequent to excluding questionnaires with inadequate completion time, a pool of 606 valid responses was retained, thus yielding an effective recovery rate of 92.8%.The examination of demographic characteristics from the formal survey unfolds as follows: Gender distribution revealed that 46.5% identified as male, while 53.5% identified as female. The age distribution indicated that 37.9% fell within the age bracket of 20 to 29 years old, 38.3% ranged from 30 to 39 years old, 20.5% were aged between 40 and 49 years old, and 3.3% were aged 50 years old and above. Concerning educational attainment, 38% possessed specialized or lower educational credentials, 52.1% held undergraduate degrees, and 9.9% had postgraduate qualifications. Monthly income distribution manifested as follows: 13.5% earned less than 2,000 RMB, 16.5% garnered between 2,000 and 5,000 RMB, 46.5% earned within the range of 5,000 to 8,000 RMB, and 23.5% earned above 8,000 RMB. Occupation-wise, the participants were distributed as follows: 13.5% identified as students, 17.2% as employees in the private sector, 8.9% as clerical workers, 9.6% as financial professionals, 10.6% as administrators, 8.3% as sales personnel, and 8.3% as belonging to diverse occupational categories. The collective representation of miscellaneous occupations accounted for 31.9%. The resulting statistical summary is provided in the ensuing Table 2.

**Table 2:** Descriptive statistics table.

Variable	Option	Number	Proportion
Gender	Male	282	46.5%
	Female	324	53.5%
Age	20–29 years old	230	37.9%
	30–39 years old	232	38.3%
	40–49 years old	124	20.5%
	50 years old and above	20	3.3%
Academic qualifications	Specialized and below	230	38.0%
	Undergraduate	316	52.1%
	Master	60	9.9%
Monthly salary	Under 2,000 RMB	82	13.5%
	2,000–5,000 RMB	100	16.5%
	5,000–8,000 RMB	282	46.5%
	More than 8,000 RMB	142	23.5%
Career	Students	82	13.5%
	Private sector workers	104	17.2%
	Clerical workers	54	8.9%
	Financial workers	58	9.6%
	Administrative workers	64	10.6%
	Sales workers	50	8.3%
	Other occupations	194	31.9%

### 4.2. Data validity test

#### 4.2.1. Common method deviation test

Given that all variables under investigation in this study were assessed from the same pool of participants, it is crucial to acknowledge the potential presence of common method bias. In order to address this concern, a Harman one-way test was conducted, aiming to assess the extent of shared variance attributable to a single methodological factor. The analysis revealed that the explained variance of the first principal component amounted to 38.66%. Importantly, this value did not surpass the critical threshold of 40%, thus indicating an absence of significant common method bias issues within the present study.

#### 4.2.2. Confidence validity test

The first step involved an assessment of the questionnaire’s reliability utilizing Cronbach’s α coefficient method. The overall Cronbach’s α value was determined to be 0.906, surpassing the critical threshold of 0.7. This outcome indicates that the measurement scale exhibited favorable reliability. Specifically, from Table 3 the Cronbach’s α values for information capability, green trust, media publicity, and green consumption intention were 0.840, 0.832, 0.811, and 0.834, respectively. Each of these values exceeded the critical threshold of 0.7, further confirming the satisfactory reliability of the respective scales. Subsequently, validity assessments were conducted. KMO value exceeding 0.8 and a value of p indicating significance for Bartlett’s test (with a threshold of 0.05) were used as criteria for evaluating scale validity. In Table 4, the KMO value was calculated as 0.895, and the value of p was determined to be 0.000. These results indicate strong validity and support the suitability of the scale for subsequent model testing.

**Table 3:** Reliability test.

	Information capability	Green trust	Media publicity	Green consumption intention
Cronbach’s α	0.840	0.832	0.811	0.834

**Table 4:** Validity test.

	KMO and Bartlett’s test	
KMO value		0.895
	Approximate cardinality	5165.824
Bartlett test	df	153
	*p* value	0.000

#### 4.2.3. Validation factor analysis

The scales underwent a validation factor analysis, and the corresponding results are presented in Table 5. The evaluation of model fit yielded the following values: *χ*^2^/df = 1.961, GFI = 0.959, TLI = 0.971, CFI = 0.977, and RMSEA = 0.04. In accordance with the established criteria, a model is considered to have a good fit when *χ*^2^/df is below 3, GFI exceeds 0.9, TLI surpasses 0.9, CFI is above 0.9, and RMSEA falls below 0.05. Based on the aforementioned fit indicators, the overall model exhibited a favorable fit. The values obtained for this study meet the fundamental requirements for model assessment, affirming that the proposed model aligns well with the observed data.

**Table 5:** Validation factor analysis.

	*χ*^2^/df	GFI	TLI	CFI	RMSEA
Measured value	1.961	0.959	0.971	0.977	0.040

### 4.3. Test of variability

The examination of gender as a variable revealed that, due to its binary nature with options limited to men and women, an independent sample t-test was deemed appropriate. The corresponding outcomes are presented in Table 6. The analysis indicates that the obtained value of p exceeds 0.05, signifying the absence of a significant difference between genders in terms of their willingness to engage in green consumption. Furthermore, a one-way analysis of variance (ANOVA) was employed to assess disparities in green consumption intention among various subgroups, including age, education, monthly salary, and occupation. As delineated in Table 7, statistically significant differences (*p* < 0.05) were observed in the intention to consume green across different education levels and monthly salary brackets. Conversely, the *p*-values associated with age and occupation surpassed 0.05, indicating a lack of statistical significance and thus no discernible differences in green consumption intention within these variables.

**Table 6:** *T*-test analysis results.

Analysis	Gender	(Mean ± standard deviation)	*p*
	Male (*n* = 282)	Female (*n* = 324)	
Result	3.336 ± 1.050	3.376 ± 1.079	0.638

**Table 7:** Results of single factor analysis.

Analysis	Sum of squares		Degree of freedom *df*		*F*	*p*
	Intergroup	Within group	intergroup	Within group		
Age	7.949	678.363	3	602	2.351	0.071
Academic qualifications	26.907	659.405	2	603	12.303	0.000
Monthly salary	16.234	670.078	3	602	4.861	0.002
Career	21.965	664.347	12	593	1.634	0.078

### 4.4. Examination of the association between information capability, green trust, and green consumption intention

To examine the relationship between information capability and green consumption intention, this study adopts a regression model with green consumption intention serving as the dependent variable. The model incorporates both the independent variable (information capability) and a control variable in sequential order. The findings for Model 4, as presented in Table 8, indicate that the β coefficient for the impact of information capability on green consumption intention is 0.587 (*p* < 0.001), reaching the threshold of statistical significance. Consequently, hypothesis H1 is supported, affirming a significant positive effect of information capability on consumers’ inclination toward green consumption. The results of the analysis suggest that information capability plays a pivotal role in influencing behavioral changes, particularly within the context of the digital information era (Wu Q. et al., 2022). Consumers possessing a strong information capability exhibit a heightened mastery of green information. Thus, information capability serves as a catalyst for enhancing green consumption intention. As a result, improving information capability becomes an imperative requirement in the digital information era, laying the foundation for promoting a shift toward more environmentally conscious consumption patterns.

**Table 8:** Hierarchical regression results.

Variables	Green trust			Green consumption intention	
	Model 1	Model 2	Model 3	Model 4	Model 5
*Control variables*					
Gender	−0.004	−0.032	0.021	−0.013	−0.006
Age	−0.043	−0.032	0.041	0.054	0.061
Academic qualifications	−0.120**	−0.043	−0.190***	−0.097**	−0.089**
Monthly salary	0.052	0.052	0.085	0.085*	0.074*
Career	0.033	−0.012	−0.003	−0.051	−0.049
*Independent variables*					
Information capability	——	0.491***	——	0.587***	0.487***
*Intermediate variables*					
Green trust	——	——	——	——	0.205***
*R* ^2^	0.025	0.257	0.037	0.370	0.401
*F*	3.071	34.593	4.669	58.511	57.096

* represents *p* < 0.05, ** represents *p* < 0.01, *** represents *p* < 0.001, same below.

To investigate the mediating role of green trust, this study constructs regression models incorporating green trust as the dependent variable and sequentially includes a control variable and the independent variable (information capability). This process results in the development of Model 1 and Model 2, as displayed in Table 8. Additionally, by considering green consumption intention as the dependent variable, the regression models include the control variable, independent variable (information capability), and mediating variable (green trust). This leads to the creation of Model 3, Model 4, and Model 5, as depicted in Table 8. The outcomes of the regression analysis for the mediating model are presented in Table 8. It is observed that information capability significantly and positively influences green trust (β = 0.491, *p* < 0.001). After including both information capability and green trust in the regression model for green consumption intention, the effects of both variables on green consumption intention remain significant. Notably, the standardized coefficient of information capability on consumers’ green consumption intention diminishes from 0.587 to 0.487 upon the inclusion of the mediating variable (green trust). Consequently, it can be inferred that green trust partially mediates the relationship between information capability and green consumption intention, providing initial support for hypothesis H2. The findings demonstrate that green trust serves as a crucial link in the process by which information capability influences consumers’ green consumption intention. On one hand, consumers’ elevated information capability contributes to increased knowledge regarding green products and recognition of their environmental benefits, thereby fostering green trust. On the other hand, green trust heightens consumers’ expectations for eco-friendly products and motivates companies to enhance their technology, minimize environmental impact, and improve their green brand image (Chen, 2010). Consequently, consumers are more inclined to select environmentally conscious products, thereby augmenting their willingness to engage in green consumption.

To further examine the mediation effect, the PROCESS plug-in was employed, utilizing a Bootstrap approach with 5,000 samples to construct 95% confidence intervals. This facilitated the calculation of direct and indirect effects under the mediation condition. The test results are presented in Table 9.

**Table 9:** Bootstrap analysis of mediating effects.

Path	Effect	Boot SE	Boot LLCI	Boot ULCI
Information capability → Green trust → Green consumption intention	0.147	0.027	0.095	0.198
Direct effect	0.684	0.051	0.583	0.784
Total effect	0.830	0.045	0.741	0.920

From Table 9, it is evident that the 95% confidence interval for the direct effect of information capability on green consumption intention, denoted as [0.583, 0.784], does not encompass zero. This finding signifies a significant direct effect. Moreover, the 95% confidence interval for the path representing information capability → green trust → green consumption intention, indicated as [0.095, 0.198], also excludes zero, indicating a significant mediating effect. Consequently, it can be concluded that consumer information capability exerts both a substantial direct impact and an indirect effect on green consumption intention. Thus, information capability serves as a partial mediator for green consumption intention, further substantiating hypothesis H2.

### 4.5. Examination of the moderating effect of media publicity

To examine the moderating effect, this study first standardizes the variables of information capability and media publicity and constructs the interaction term. Subsequently, the regression model incorporates the control variable, independent variable (information capability), moderating variable (media publicity), and the interaction term. The resulting models, as presented in Table 10 from Model 1 to Model 4, allow us to observe the moderating effect of the interaction term between information capability and media publicity on green consumption intention. The analysis reveals that the interaction term of information capability and media publicity exhibits a significant moderating effect on green consumption intention, as indicated by a beta value of 0.085 (*p* < 0.05). This finding signifies that media publicity plays a reinforcing moderating role in shaping the relationship between information capability and consumers’ green consumption intention. This phenomenon can be attributed to the inherent information uncertainty prevalent in the digital information age (Ferracuti, 2022). Furthermore, within a context of high media advocacy, consumers are exposed to elevated levels of knowledge regarding green information, thereby validating the authenticity of a product’s green attributes (Tony et al., 2022). Consequently, this validation process reinforces consumers’ motivation, particularly those with high information capability, to exhibit a greater willingness to engage in green consumption practices.

**Table 10:** Results of the moderating effect test.

Variables	Green consumption intention			
	Model 1	Model 2	Model 3	Model 4
*Control variables*				
Gender	0.021	−0.013	−0.005	−0.014
Age	0.041	0.054	0.062	0.058
Academic qualifications	−0.190***	−0.097**	−0.105**	−0.099**
Monthly salary	0.085	0.085*	0.104**	0.102**
Career	−0.003	−0.051	−0.055	−0.053
Independent variable				
Information capability	——	0.587***	0.457***	0.419***
Moderating variable				
Media publicity	——	——	0.232***	0.239***
*Interaction item*				
Information capability × Media publicity	——	——	——	0.085*
*R* ^2^	0.037	0.370	0.406	0.412
*F*	4.669	58.511	58.447	52.283

* represents *p* < 0.05, ** represents *p* < 0.01, *** represents *p* < 0.001.

To further examine the moderating effect, the PROCESS plug-in was employed to assess the results of the moderating effect, as presented in Table 11. Analysis of Table 11 reveals a significant interaction between information capability and media publicity on green consumption intention. The 95% confidence intervals, namely (0.612, 0.841) and (0.301, 0.646), demonstrate that these intervals do not encompass 0, thus indicating a positive moderating effect of media publicity on the relationship between information capability and green consumption intention. Specifically, the confidence intervals of (0.612, 0.841) and (0.301, 0.646) correspond to the moderating effect on the information capability → green consumption intention path.

**Table 11:** Results of moderating effects.

Variable	Effect	Boot SE	Boot LLCI	Boot ULCI	Significance
High media publicity	0.726	0.058	0.612	0.841	***
Low media publicity	0.473	0.088	0.301	0.646	***

**Figure 2:**
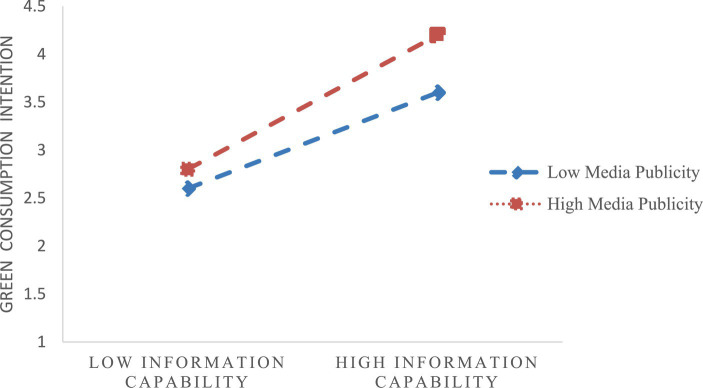
Moderating role of media publicity.

Figure 2 illustrates the slope diagram depicting the moderating effect of media publicity. The figure indicates that media publicity effectively regulates the relationship between information capability and green consumption intention. When media publicity is higher, it amplifies the positive impact of information capability on consumers’ green consumption intention. Conversely, when media publicity is lower, it diminishes the positive impact of information capability on consumers’ green consumption intention.

## 5. Discussion

### 5.1. Research findings

From the vantage point of information demand and rooted in information asymmetry theory and cognitive bias theory, this paper constructs a model of the mechanism of the influence of consumers’ information capability on green consumption willingness, and explores the mediating role of consumers’ green trust and the moderating role of media publicity. This paper advances a series of hypotheses grounded in theoretical scrutiny. Subsequently, utilizing questionnaire data, the proposed hypotheses are subjected to empirical scrutiny employing methodologies encompassing hierarchical regression and other empirical techniques. To reinforce the assessment of mediation and moderation effects, advanced methods such as Bootstrap are employed for validation. These analytical approaches yield the ensuing conclusions.

The findings of this study reveal several significant outcomes. Firstly, it is observed that information capability exerts a substantial positive influence on green consumption intention, confirming the influence of information capability on green behavior (Lv and Li, 2023).This indicates that consumers’ cognitive ability plays a pivotal role in shaping their inclination toward environmentally conscious consumption. Specifically, consumers with higher cognitive abilities demonstrate enhanced knowledge about green products and possess greater efficiency in interpreting information. Consequently, they exhibit a heightened willingness to adopt green consumption practices. It also further illustrates the existence of cognitive constraints (Matteo et al., 2020); Moreover, the study identifies that information acquisition capability serves as a crucial factor in mitigating information asymmetry and reducing market uncertainties. Furthermore, the study highlights the positive impact of information sharing capability on green consumption intention. This signifies that consumers who possess a certain level of understanding regarding green products and sustainable consumption engage in information exchange with their social networks, thus facilitating information flow, addressing information asymmetry challenges, improving information availability, deepening comprehension of green consumption, and fostering the influence of subjective norms. Consequently, this social influence mechanism generates a “herd effect” (Sterenn et al., 2018) and a “demonstration effect” (Hu et al., 2022), stimulating changes in consumption patterns and elevating consumers’ willingness to embrace green products, even among those who have not yet adopted green consumption practices.

Secondly, the study identifies the mediating role of green trust in the relationship between information capability and green consumption intention, supporting Hashish et al.’s (2022), view that whether consumers engage in green behaviors or not depends largely on the role of green trust. It is observed that as consumers’ information capability strengthens, their knowledge about green products and the broader environmental context increases, leading to the development of trust in the efficacy of green products and the commitment of companies toward sustainability. Trust, being a crucial prerequisite for consumer willingness, plays a pivotal role in reducing apprehensions and fostering belief in the positive outcomes of green products for both individual well-being and the external environment. Consequently, this enhanced trust facilitates the adoption of green consumption practices and elevates consumer willingness. Hence, it can be inferred that consumer information capability indirectly influences green consumption intention by fostering the development of green trust as an intermediary mechanism.

Lastly, it is found that media publicity serves as a positive moderator in the direct relationship between information capability and consumers’ green consumption intention. In the digital age, media assumes a significant role as a primary channel for information dissemination. Companies utilize media platforms to promote the environmentally friendly features of their products, thereby enhancing the authenticity of their green brands. Consequently, when consumers engage in the purchase of green products, they associate the product’s sustainable contributions to environmental preservation and embrace its inherent green value. Notably, heightened media publicity acts as a catalyst, particularly for consumers possessing high levels of information capability, further augmenting their inclination toward adopting green consumption practices.

### 5.2. Theoretical contributions

The present study makes several theoretical contributions. Firstly, it sheds light on the concept of information capability, which serves as a novel imperative for consumers in the digital information era. Despite its significance, limited research has explored this area comprehensively. Consequently, this paper fills this gap by adopting an innovative approach and constructing a model that investigates the impact of information capability on consumers’ green consumption intention. By examining information capability as the focal point, this research provides valuable insights into consumer behavior and contributes to the existing body of knowledge in this field.

Secondly, this study delves into the influence of information capability on green consumption intention by incorporating additional variables to enhance understanding. Specifically, green trust and media publicity are introduced as mediating and moderating variables, respectively, in order to investigate the underlying mechanisms in greater depth. By considering these factors alongside the direct effects, this research offers a more comprehensive analysis of the impact of information capability on green consumption intention in the digital information era. Through this theoretical and empirical exploration, it provides a novel explanatory perspective and valuable empirical evidence, enriching the understanding of how information capability shapes consumers’ inclination toward green consumption.

### 5.3. Research recommendations

Firstly, it is imperative for both governmental institutions and enterprises to intensify efforts in promoting and disseminating knowledge pertaining to green consumption and green products. This involves facilitating the transfer of information from the supply side to the demand side, enhancing consumers’ cognitive abilities and knowledge levels, and creating favorable conditions that optimize the efficacy of information transfer mechanisms. Concurrently, the government demonstrate a keen awareness of the constraints and disparities inherent in consumers’ information cognitive capability. Accordingly, they employ strategic measures to craft informational content and formats that facilitate enhanced comprehension and cognition. This approach is taken with the aim of circumventing the adoption of overly simplistic and rudimentary tactics typified by “information bombardment.” Instead, the government endeavors to disseminate knowledge related to green consumption and its associated ecological significance in a manner that optimizes effectiveness and accessibility. Additionally, the government should enhance accessibility to information on green products, leveraging various information channels, particularly computer and cellphone networks, which are readily accessible and frequently used by consumers. This approach effectively addresses the “information dilemma” and alleviates decision-making anxiety, empowering consumers to actively, purposefully, and effectively obtain information. Furthermore, the government should establish robust information exchange channels, allowing consumers to share information, deepen their understanding of green consumption, and enhance their willingness to engage in green practices. Enterprises, on the other hand, should cultivate the image of being green-focused organizations and foster green brands that elucidate the environmental contributions of their products. By augmenting the perceived value of green attributes and bolstering consumers’ green trust, enterprises can foster a greater propensity for green consumption. Moreover, media platforms should be utilized to publicize the green attributes of products, enhance their authenticity, and amplify consumers’ willingness to engage in green consumption. Secondly, consumers themselves must proactively enhance their information capabilities by cultivating a greater efficiency in interpreting information. They should employ diverse methods to acquire information, ensuring its accuracy, and augmenting their own information capabilities.

### 5.4. Research limitations and outlook

The limitations of this study are as follows: Primarily, this study initiated its investigation from the consumer perspective, focusing on the information demand aspect. It incorporated consumer green trust as a mediating variable and media publicity as a moderating variable to unravel the route through which consumers’ information competence shapes their propensity for green consumption. However, it’s important to acknowledge that the current model’s explanatory capacity might hold some limitations. Subsequent research endeavors could consider integrating psychological factors like perceived ease of use as mediating variables, thereby augmenting the model’s explanatory prowess. Secondly, this study’s presumptions about information predominantly revolved around positive information scenarios. However, the coexistence of both positive and negative information in real-world contexts could potentially introduce a bias in the analysis pertaining to the mechanism whereby information competence influences green trust and consequently, green consumption intention. To mitigate this potential bias, forthcoming research could incorporate robust methods of identification and measurement, thereby addressing this issue comprehensively. Lastly, with regard to data collection, the study employed online questionnaires within a concurrent timeframe, thereby posing challenges in establishing a causal relationship within the model. Future research could address this concern by employing methodologies such as time series design and alternative assessment approaches to collect data. These identified limitations stand as focal points for future research endeavors, prompting more comprehensive investigations in the field.

## Data availability statement

The raw data supporting the conclusions of this article will be made available by the authors, without undue reservation.

## Ethics statement

Ethical review and approval was not required for the study on human participants in accordance with the local legislation and institutional requirements. Written informed consent from the participants was not required to participate in this study in accordance with the national legislation and the institutional requirements.

## Author contributions

G-LL: conceptualization, methodology, validation, investigation, writing — review and editing, and supervision. HZ: conceptualization, validation, writing — original draff, writing — review and editing, formal analysis, and methodology. Y-LG: visualization, writing — review and editing, and validation. All authors reviewed and approved this article for publication.

## Funding

This study is partially supported by Social Science Planning Programs of Shandong Province (21DJJJ16). Research on the spatial and temporal network structure and influence mechanism of foreign trade in Shandong Province under the background of international trade friction.

## Footnotes

^1^ https://www.wjx.cn

## References

[ref1] Adrian R. C. Ben R. N. (2013). Mind the gap? Description, experience, and the continuum of uncertainty in risky choice. Prog. Brain Res. 202, 55–71. doi: 10.1016/B978-0-444-62604-2.00004-6, PMID: 23317826

[ref2] Akhtar R. Sultana S. Masud M. M. Jafrin N. Al M. A. (2020). Consumers’ environmental ethics, willingness, and green consumerism between lower and higher income groups. Resour. Conserv.Recy. 168:105274. doi: 10.1016/j.resconrec.2020.105274

[ref3] Albert B. (2012). On the functional properties of perceived self-efficacy revisited. J. Manage. 38, 9–44. doi: 10.1177/0149206311410606

[ref4] Allari R. S. Hamdan K. Albqoor M. A. Shaheen A. (2022). Information literacy: assessment of undergraduate and graduate nursing students. Ref. Serv. Rev. 50, 211–221. doi: 10.1108/RSR-09-2021-0052

[ref5] Bartschat M. Cziehso G. Hennig-Thurau T. (2022). Searching for word of mouth in the digital age: determinants of consumers’ uses of face-to-face information, internet opinion sites, and social media. J. Bus. Res. 141, 393–409. doi: 10.1016/j.jbusres.2021.11.035

[ref6] Behrens S. J. (1994). A conceptual analysis and historical overview of information literacy. Coll. Res. Libr. 55, 309–322. doi: 10.5860/crl_55_04_309

[ref7] Brian S. Martin G. Benjamin D. Gilbert S. (2015). Assessing the value and role of seafood traceability from an entire value-chain perspective. Compr. Rev. Food Sci. Food Saf. 14, 205–268. doi: 10.1111/1541-4337.12130, PMID: 33401795

[ref8] Carsten D. Sinne S. Laura M. A. Yonatan S. (2014). Improving eco-labelling as an environmental policy instrument: knowledge, trust and organic consumption. J. Environ. Pol. Plan. 16, 559–575. doi: 10.1080/1523908X.2013.879038

[ref9] Cavaliere A. Crea G. (2021). Brand premia driven by perceived vertical differentiation in markets with information disparity and optimistic consumers. J. Econ. 135, 223–253. doi: 10.1007/S00712-021-00761-9

[ref10] Chaihanchanchai P. Anantachart S. (2022). Encouraging green product purchase: green value and environmental knowledge as moderators of attitude and behavior relationship. Bus. Strateg. Environ. 32, 289–303. doi: 10.1002/BSE.3130

[ref11] Chen Y. (2010). The drivers of green brand equity: green brand image, green satisfaction, and green trust. J. Bus. Ethics 93, 307–319. doi: 10.1007/s10551-009-0223-9

[ref12] Courtade T. Grama A. Mahoney M. W. Weissman T. (2017). Principles and applications of science of information. Proc. IEEE 105, 183–188. doi: 10.1109/jproc.2016.2646778

[ref13] Di M. Nanere Souza D. (2019). The effect of pro-environmental attitudes and eco-labelling information on green purchasing decisions in Australia. J. Nonprofit. Public. Sec. Mark. 31, 201–225. doi: 10.1080/10495142.2019.1589621

[ref14] Dongfang G. (2021). Flagging fake news on social media: an experimental study of media consumers' identification of fake news. Gov. Inform. Q. 38:101591. doi: 10.1016/J.GIQ.2021.101591

[ref15] Feng W. Zeng Y. Shen X. Liu C. (2022). Green advertising is more environmentally friendly? The influence of advertising color on consumers’ preferences for green products. Front. Psychol. 13:959746. doi: 10.3389/FPSYG.2022.959746, PMID: 36389554 PMC9648352

[ref16] Ferracuti E. (2022). Information uncertainty and organizational design. J. Account. Econ. 74:101493. doi: 10.1016/J.JACCECO.2022.101493

[ref17] Gabi E. (2020). Individual initiative and burnout as antecedents of employee expediency and the moderating role of conscientiousness. J. Bus. Res. 110, 202–212. doi: 10.1016/j.jbusres.2019.12.047

[ref18] Hashish M. E. Abdou A. H. Mohamed S. A. K. Elenain A. S. A. Salama W. (2022). The Nexus between green perceived quality, green satisfaction, green trust, and customers’ green behavioral intentions in eco-friendly hotels: a structural equation modeling approach. Int. J. Env. Res. Pub. Health 19:16195. doi: 10.3390/IJERPH192316195, PMID: 36498268 PMC9736449

[ref19] Hossain I. Nekmahmud M. FeketeFarkas M. (2022). How do environmental knowledge, eco-label knowledge, and green trust impact consumers’ pro-environmental behaviour for energy-efficient household appliances? Sustainability 14:6513. doi: 10.3390/SU14116513

[ref20] Hu D. Gu Q. Zhang Y. (2022). Role modeling effects: how leader's job involvement affects follower creativity. Asia. Pac. J. Hum. Resour. 61, 101–123. doi: 10.1111/1744-7941.12332

[ref21] Hung V. N. Cuong H. N. Thoa T. B. H. (2019). Green consumption: closing the intention-behavior gap. Sustain. Dev. 27, 118–129. doi: 10.1002/sd.1875

[ref22] Jin M. Li B. Xiong Y. Chakraborty R. Zhou Y. (2023). Implications of coproduction technology on waste management: who can benefit from the coproduct made of leftover materials? Eur. J. Oper. Res. 307, 1248–1259. doi: 10.1016/J.EJOR.2022.10.020

[ref23] Kahneman D. Tversky A. (1979). On the interpretation of intuitive probability: a reply to Jonathan Cohen. Cognition 7, 409–411. doi: 10.1016/0010-0277(79)90024-6

[ref24] Kaman L. (2010). The green purchase behavior of Hong Kong young consumers: the role of peer influence, local environmental involvement, and concrete environmental knowledge. J. Int. Consum. Mark. 23, 21–44. doi: 10.1080/08961530.2011.524575

[ref25] Khatskevich V. L. (2022). Means of fuzzy numbers in the fuzzy information evaluation problem. Automat. Rem. Contr. 83:407. doi: 10.1134/S0005117922030080

[ref26] Laura P. Kevin Z. Z. Julie J. L. (2016). When can you trust “trust”? Calculative trust, relational trust, and supplier performance. Strateg. Manage. 37:724. doi: 10.1002/smj.2374

[ref27] Li Y. Quan D. Zhang H. (2020). Linking consumers' anticipated guilt to green consumption intention: testing the role of perceived consumer effectiveness and green involvement. Int. J. Environ. Pollut. 68, 145–161. doi: 10.1504/IJEP.2020.120175

[ref28] Liu J. Hou Y. Wang J. Fu P. Xia C. (2022). How does leaders' information-sharing behavior affect subordinates' taking charge behavior in public sector? A moderated mediation effect. Front. Psychol. 13:938762. doi: 10.3389/FPSYG.2022.938762, PMID: 36570996 PMC9768551

[ref29] Luan J. Filieri R. Xiao J. Han Q. Zhu B. Wang T. (2023). Product information and green consumption: an integrated perspective of regulatory focus, self-construal, and temporal distance. Inform. Manage-Amster. 60:103746. doi: 10.1016/J.IM.2022.103746

[ref30] Lv X. Li J. (2023). A benchmark model for exploring the differentiation of trust in information sources in heterogeneous farmers' green behavior. Environ. Sci. Pollut. R. 30, 69941–69954. doi: 10.1007/S11356-023-27340-3, PMID: 37142840

[ref31] Manoela C. P. Edvan C. A. (2020). How self-expressive benefits relate to buying a hybrid car as a green product. J. Clean. Prod. 252:119859. doi: 10.1016/j.jclepro.2019.119859

[ref32] Martin H. T. (2015). Maintaining trust and credibility in a continuously evolving organic food system. J. Agr. Environ. Ethic. 28, 767–787. doi: 10.1007/s10806-015-9559-6

[ref33] Matteo P. Carl M. Andreea M. (2020). Information avoidance and overvaluation under epistemic constraints: principles and implications for regulatory policies. Reliab. Eng. Syst. Safe. 197:106814. doi: 10.1016/j.ress.2020.106814

[ref34] Mcclure C. R. (1994). Network literacy: a role for libraries? Inform. Technol. Libr. 5, 180–190. doi: 10.1287/isre.5.2.180

[ref35] Michael R. D. Edi K. (1973). Free competition and the optimal amount of fraud. J. Law Econ. 16, 67–88. doi: 10.1086/466756

[ref36] Muddasar G. K. Saqib M. Ahmad J. (2020). Online information bombardment! How does eWOM on social media lead to consumer purchase intentions? Int. J. Grid. Util. Comp. 11, 857–867. doi: 10.1504/IJGUC.2020.110918

[ref37] Nancy M. F. (2015). From standards to frameworks for IL: how the ACRL framework addresses critiques of the standards. Port. Libr. Acad. 15, 699–717. doi: 10.1353/pla.2015.0045

[ref38] Niedermeier A. Emberger K. A. Menrad K. (2020). Drivers and barriers for purchasing green fast-moving consumer goods: a study of consumer preferences of glue sticks in Germany. J. Clean. Prod. 284:124804. doi: 10.1016/j.jclepro.2020.124804

[ref39] Nisha P. K. Sherry A. J. Edmund P. (2016). Examining the impact of socialization and information sharing and the mediating effect of trust on innovation capability. Int. J. Oper. Prod. Man. 36, 1601–1624. doi: 10.1108/IJOPM-09-2015-0558

[ref40] Otterbring T. Sundgot B. C. Bratland S. S. Trangsrud L. K. J. (2023). Siblings, shopping, and sustainability: birth-order differences in green consumption. Front. Psychol. 14:1105072. doi: 10.3389/FPSYG.2023.1105072, PMID: 36935953 PMC10020628

[ref41] Ramesh K. Raiswa S. Sekar P. C. Richa D. (2019). Examining the role of external factors in influencing green behaviour among young Indian consumers. Young Consum. 20, 380–398. doi: 10.1108/YC-12-2018-0921

[ref42] Ran Y. Nilsson L. A. Dawkins E. Grah R. Vanhuyse F. Engström E.. (2022). Information as an enabler of sustainable food choices: a behavioural approach to understanding consumer decision-making. Sustain. Prod. Consum. 31, 642–656. doi: 10.1016/J.SPC.2022.03.026

[ref43] Razzaq A. Tang Y. Qing P. (2021). Towards sustainable diets: understanding the cognitive mechanism of consumer acceptance of biofortified foods and the role of nutrition information. Int. J. Env. Res. Pub. Health 18:1175. doi: 10.3390/IJERPH18031175, PMID: 33525742 PMC7908173

[ref44] Septianto F. Kemper J. A. (2021). The effects of age cues on preferences for organic food: the moderating role of message claim. J. Retail. Consum. Serv. 62:102641. doi: 10.1016/J.JRETCONSER.2021.102641

[ref45] Siti M. Kian Y. K. Seethaletchumy T. (2020). Factors influencing non-green consumers' purchase intention: a partial least squares structural equation modeling (PLS-SEM) approach. J. Clean. Prod. 280:124192. doi: 10.1016/j.jclepro.2020.124192

[ref46] So-Yun K. Jungsung Y. Sang H. S. Suhyun S. (2012). Toward a composite measure of green consumption: an exploratory study using a Korean sample. J. Fam. Econ. Iss. 33, 199–214. doi: 10.1007/s10834-012-9318-z

[ref47] Sterenn L. Frédéric S. Dorothée B. (2018). Green consumption and peer effects: does it work for seafood products? Food Policy 76, 44–55. doi: 10.1016/j.foodpol.2018.02.017

[ref48] Tong L. Toppinen A. Wang L. Berghäll S. (2023). How motivation, opportunity, and ability impact sustainable consumption behaviour of fresh berry products. J. Clean. Prod. 401:136698. doi: 10.1016/J.JCLEPRO.2023.136698

[ref49] Tony W. Agung U. Anita M. Zahrotush S. Punitha S. (2022). Motives and barriers of organic food consumption behaviour: a comparative study between Indonesia and Malaysia. Int. J. Green. Econ. 16, 1–17. doi: 10.1504/IJGE.2022.10048654

[ref50] Wang J. Wangt C. (2022). Analysis of airbnb’s green user emotional characteristics: how do human, geographical, housing, and environmental factors influence green consumption? Front. Env. Sci. Switz. 10:993677. doi: 10.3389/FENVS.2022.993677

[ref51] Wu Q. Gao S. Wang X. Zhao Y. (2022). Research on the impacts of information capacity on farmers’ green prevention and control technology adoption. Ecol. Chem. Eng. S. 29, 305–317. doi: 10.2478/ECES-2022-0022

[ref52] Wu S. Sun H. Liu M. (2022). Research on the influencing factors of proactive green innovation in manufacturing enterprises. Ind Eng Innov Manag. 5:050107. doi: 10.23977/IEIM.2022.050107

[ref53] Wu L. Wang S. Zhu D. Hu W. Wang H. (2015). Chinese consumers' preferences and willingness to pay for traceable food quality and safety attributes: the case of pork. China Econ. Rev. 35, 121–136. doi: 10.1016/j.chieco.2015.07.001

[ref54] Xu A. Wei C. Zheng M. Sun L. Tang H. (2022). Influence of perceived value on repurchase intention of green agricultural products: from the perspective of multi-group analysis. Sustainability 14:15451. doi: 10.3390/SU142215451

[ref55] Yang X. Kittikowit S. Noparumpa T. Jiang J. Chen S. C. (2021). Moderated mediation mechanism to determine the effect of gender heterogeneity on green purchasing intention: from the perspective of residents' values. Front. Psychol. 12:803710. doi: 10.3389/fpsyg.2021.803710, PMID: 35145461 PMC8823626

[ref56] Yudi F. Ahmed Z. A. Muhammad S. S. (2020). The nexus of information sharing, technology capability and inventory efficiency. J. Glob. Oper. Strateg. 33, 327–351. doi: 10.1108/JGOSS-02-2020-0011

[ref57] Zhao H. Gao Q. Wu Y. Zhu X. (2014). What affects green consumer behavior in China? A case study from Qingdao. J. Clean. Prod. 63, 143–151. doi: 10.1016/j.jclepro.2013.05.021

[ref58] Zhao M. Steve H. Gal Z. (2011). Mental simulation and product evaluation: the affective and cognitive dimensions of process versus outcome simulation. J. Mark. Res. 48, 827–839. doi: 10.1509/jmkr.48.5.827

[ref59] Zrnec A. Poženel M. Lavbič D. (2022). Users’ ability to perceive misinformation: an information quality assessment approach. Inform. Process. Manag. 59:102739. doi: 10.1016/J.IPM.2021.102739

